# The Histone Acetyltransferase CfGcn5 Regulates Growth, Development, and Pathogenicity in the Anthracnose Fungus *Colletotrichum fructicola* on the Tea-Oil Tree

**DOI:** 10.3389/fmicb.2021.680415

**Published:** 2021-06-23

**Authors:** Shengpei Zhang, Yuan Guo, Siqi Chen, He Li

**Affiliations:** ^1^College of Forestry, Central South University of Forestry and Technology, Changsha, China; ^2^Key Laboratory of National Forestry, Grassland Administration on Control of Artificial Forest Diseases and Pests in South China, Changsha, China; ^3^Hunan Provincial Key Laboratory for Control of Forest Diseases and Pests, Changsha, China; ^4^Key Laboratory for Non-wood Forest Cultivation and Conservation of Ministry of Education, Changsha, China

**Keywords:** histone acetyltransferase, conidiation, pathogenicity, nucleus, localization, *C. fructicola*

## Abstract

The tea-oil tree (*Camellia oleifera* Abel.) is a commercial edible-oil tree in China, and anthracnose commonly occurs in its plantations, causing great losses annually. We have previously revealed that CfSnf1 is essential for pathogenicity in *Colletotrichum fructicola*, the major pathogen of anthracnose on the tea-oil tree. Here, we identified CfGcn5 as the homolog of yeast histone acetyltransferase ScGcn5, which cooperates with ScSnf1 to modify histone H3 in *Saccharomyces cerevisiae*. Targeted gene deletion revealed that CfGcn5 is important in fungi growth, conidiation, and responses to environmental stresses. Pathogenicity assays indicated that CfGcn5 is essential for *C. fructicola* virulence both in unwounded and wounded tea-oil tree leaves. Further, we found that CfGcn5 is localized to the nucleus and this specific localization is dependent on both NLS region and HAT domain. Moreover, we provided evidence showing that the nuclear localization is essential but not sufficient for the full function of CfGcn5, and the NLS, HAT, and Bromo domains were proven to be important for normal CfGcn5 functions. Taken together, our studies not only illustrate the key functions of CfGcn5 in growth, development, and pathogenicity but also highlight the relationship between its locations with functions in *C. fructicola*.

## Introduction

The tea-oil tree (*Camellia oleifera* Abel.) is a commercial shrub native to China and has been widely grown in southern China for over 2000 years (Chen et al., 2015). The tea oil extracted from its seed, similar to olive oil, is an excellent edible oil for humans and popular in Chinese cooking (Feas et al., 2013; Di et al., 2018). Though plantations have increased to more than 3.7 million hectares and tea oil yield ranges from 450 to 750 kg/ha, it is still not enough for the demand (Chen et al., 2015). Unfortunately, the tea-oil tree can be infected by numerous serious diseases which limit its cultivation.

Anthracnose is the most devastating disease afflicting the tea-oil tree and commonly occurs in plantations (Li et al., 2016). All tea-oil tree tissues are susceptible to the disease, resulting in the constant dropout of leaves and fruit. Anthracnose is currently causing a 10–30% reduction in tea oil each year, and some areas often incur over 50% tea oil losses. *Colletotrichum*, the causal agent of anthracnose, is one of the top 10 plant-pathogenic fungi and almost every crop is susceptible to one or more species of such a genus (Dean et al., 2012). For a long time, the *Colletotrichum gloeosporioides* complex has been considered as the pathogen of anthracnose on the tea-oil tree. Whereas, we recently revealed that its pathogens include at least *C. gloeosporioides*, *C. fructicola*, *C. siamense*, *C. karstii*, and *C. camelliae*, and that *C. fructicola*, the widest distributed and strongest pathogenic specie, was the major pathogen (Li et al., 2016; Jiang and Li, 2018).

*C. fructicola* was first identified in coffee berries in 2009 and was distributed all over the world, which resulted in the infection of over 50 plants, such as apples, pears and strawberries, among others (Prihastuti et al., 2009; Weir et al., 2012; Rockenbach et al., 2016; Liang et al., 2018). Despite its economic and ecological importance, its molecular pathogenesis is unclear. Previous studies revealed that *C. fructicola* contains a large amount of candidate virulence genes and many of them are upregulated during early plant colonization (Baroncelli et al., 2016; Liang et al., 2018), but the functions of these putative virulence genes remain unknown.

We recently demonstrated that CfSnf1, a key component of AMPK (AMP-activated protein kinase), regulates the utilization of specific carbon sources, appressorium formation, and pathogenicity in *C. fructicola* (Zhang et al., 2019). In *Saccharomyces cerevisiae*, ScSnf1 cooperates with the histone acetyltransferase ScGcn5 (general control non-derepressible 5) to regulate transcription (Lo et al., 2001; Abate et al., 2012). ScGcn5 was the first HAT (histone acetyltransferase) identified in yeast and contains a HAT domain and a Bromo domain (Haynes et al., 1992; Candau et al., 1997). It mainly targets specific lysine residues in histones H3 and H2B, and thus epigenetically regulates global gene transcription to environmental adaption (Zhang et al., 1998; Suka et al., 2001; Gaupel et al., 2015; Hu et al., 2015). There is little evidence supporting the importance of Gcn5 in the fungal development and virulence of phytopathogens. In *Magnaporthe oryzae*, MoGcn5 regulates the pathogenicity through acetylating the autophagy protein MoAtg7 (Zhang et al., 2017). Gcn5 proteins are also required for the development and virulence of *Ustilago maydi*s and *Fusarium graminearum* (Gonzalez-Prieto et al., 2014; Kong et al., 2018). Despite this, the function of such a protein in forest fungal pathogens including *C. fructicola* is unknown. Here, we characterized the roles of CfGcn5 in forest pathogens for the first time. And we also provided evidence demonstrating the contributions of the NLS region, HAT, and Bromo domains to localization and function by domain deletion.

## Materials and Methods

### Strains and Culture Conditions

The *C. fructicola* CFLH16 strain was used as wide-type (WT) for transformation. The WT, Δ*Cfgcn5* mutant, complemented strain Δ*Cfgcn5*/*CfGCN5*, and domain deleted strains (ΔNLS, ΔHAT, and ΔBromo) were cultured on Potato Dextrose Agar (PDA: 200 g peeled potato, 20 g dextrose, and 15 g agar in 1 L ddH_2_O) or Minimal Medium (MM: 0.52 g KCl, 6 g NaNO_3_, 1.52 g KH_2_PO_4_, 0.152 g MgSO_4_⋅7H_2_O, 0.01 g VB1, 10 g Glucose, 1 ml 1,000 × trace elements, and 15 g agar in 1 L ddH_2_O) plates at 28°C in the dark. The strains cultured in liquid PDB (Potato Dextrose Broth) for 2 or 3 days were used for DNA extraction and conidiation analysis.

### Phylogenetic Tree Construction and Domain Prediction

The Gcn5 proteins in *F. fujikuroi, F. graminearum*, *Trichoderma reesei, C. fructicola, C. gloeosporioides, M. oryzae, Neurospora crassa, Aspergillus nidulans, and S. cerevisiae* were obtained from the NCBI database^1^. The MEGA 7.0 program with the neighbor-joining method was used to construct the phylogenetic tree. The domains and NLS region of CfGcn5 were predicted by the SMART^2^ and cNLS Mapper^3^ websites, respectively.

### Gene Deletion, Complementation, and Domain Deletion Assays

Targeted *CfGCN5* gene deletion was carried out by the one-step replacement strategy as per our previous description (Zhang et al., 2019). The two ∼1.0 kb sequences flanking *CfGCN5* were overlapped to the two sides of the *HPH* (hygromycin resistance cassette) gene, respectively. Then, the ∼3.4 kb fragments were transformed into protoplasts of CFLH16 for gene deletion. For the complementation and domain deletion assays, the ∼1.5 kb native promoter and the full-length or truncated fragments (ΔNLS, or ΔHAT, or ΔBromo) of *CfGCN5* were inserted into the pYF11 (bleomycin resistance) vector as described previously (Bruno et al., 2004). After sequencing, the fused-pYF11 plasmids were transformed into protoplasts of the Δ*Cfgcn5* mutant to acquire the complemented and domain deleted strains.

### Growth, Conidiation, Germination, and Appressoria Formation Assays

The strains were cultured on PDA and MM agar plates at 28°C in the darkness for 4 days, and the colony diameters were measured and statistically analyzed. For conidiation assays, the strains were cultured in 200 mL liquid shaking PDB for 3 days, then were filtered with three layers of lens paper and the conidia were collected and quantified on a microscope. For germination and appressoria formation assays, the collected conidia were washed by ddH_2_O twice and resuspended to a concentration of 3 × 10^5^ spores/mL and inoculated onto hydrophobic artificial surfaces or an onion epidermis for germination and appressorium formation.

### Stress Response Assays

The strains were cultured on PDA and PDA plus with various stresses, including osmotic stress (1 M NaCl and 1 M KCl), cell wall integrity stress (400 μg/ml CFW, 800 μg/ml CR and 0.01% SDS), oxidative stress (2.5 mM and 5.0 mM H_2_O_2_), and ER stress (2.5 mM and 5.0 Mm DTT). After 4 days of incubation, the colony diameters were measured and the inhibition rates were statistically analyzed.

### Pathogenicity Assays

The mycelia or conidial suspensions (3 × 10^5^) of the strains were inoculated onto the edge of unwounded and wounded tea-oil leaves. For the pathogenicity on wounded apples, the apples were first punched into several 8-mm-diameter holes and the peel tissue was removed from the punched area, then 3 × 3 mm mycelia plugs were inoculated onto it. The inoculated leaves or apples were kept in a high humidity conditions at room temperature. After incubation for 4∼5 days, the lesions were observed and photographed.

### Protoplast Release Assays

The mycelia were incubated in liquid PDB for 2 days, followed by filtration and drying. For each sample, 1.0 g of dried biomass was resuspended in 0.7 M NaCl with lysing enzyme and incubated for 2 h at 30°C by gentle shaking (70 rpm). The protoplasts were observed under microscope.

### Localization Observation and DAPI Staining

The full-length and truncated CfGcn5 were fused with a green fluorescent protein (GFP) tag and transformed into the Δ*Cfgcn5* mutant. The hyphae and conidia of the strains were further stained with DAPI (50 μg/ml) for 5 min. After being washed twice with ddH_2_O, the green and blue fluorescent signals were observed under microscope.

### Statistical Analysis

All statistical data were expressed as mean ± SD and analyzed by a one-way ANOVA (Analysis of Variance) with Duncan’s new multiple range test, *P* < 0.01.

## Results

### Identification and Phylogenetic Analysis of CfGcn5 in *C. fructicola*

**FIGURE 1:**
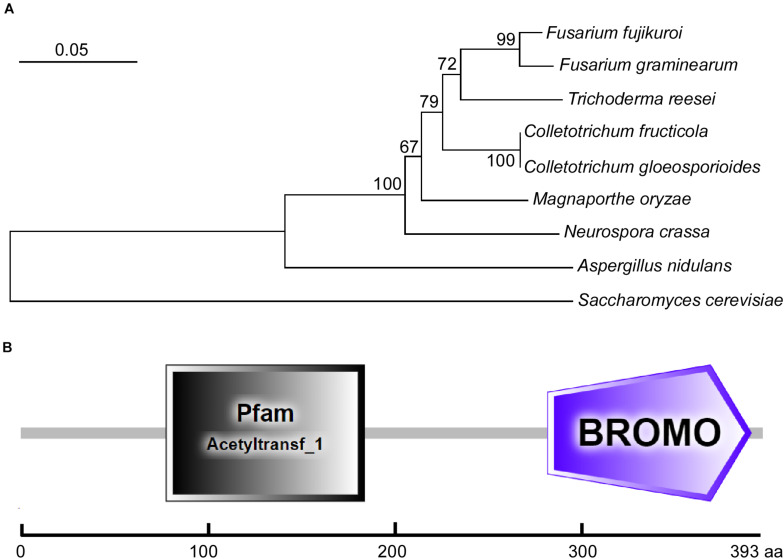
Phylogenetic analysis and domain prediction of CfGcn5. **(A)** The Gcn5 proteins from diverse fungi were aligned using CLUSTAL_W, and the phylogenetic tree was constructed using MEGA 7.0 and the neighbor-joining method with 1000 bootstrap replicates. The sequences were collected from the NCBI database and the GenBank accession numbers are shown as follows: *F. fujikuroi* (XP_023425542.1), *F. graminearum* (XP_011324943.1), *T. reesei* (XP_006966860.1), *C. fructicola* (MW701426), *C. gloeosporioides* (EQB51886.1), *M. oryzae* (XP_003716207.1), *N. crassa* (XP_001728480.2), *A. nidulans* (XP_661225.1), and *S. cerevisiae* (NP_011768.1). **(B)** The domain prediction of CfGcn5. The gray rectangle (77∼182 amino acids) indicates the acetyltransf_1 (HAT) domain and the purple pentagon (279∼387 amino acids) refers to the Bromo domain.

Using the *S. cerevisiae* ScGcn5 sequence as the trace, we identified its single homolog in the *C. fructicola* genome database by a BLAST_P search. Then, we submitted its sequence to the NCBI database (see text footnote 1) (GenBank accession number MW701426) and named it CfGcn5. We also collected the sequence of its homologs in other filamentous fungi from the NCBI database and constructed the phylogenetic tree. The phylogenetic dendrogram revealed that CfGcn5 shows sequence conservation among other fungi Gcn5 proteins; with CfGcn5 being most similar to that of *C. gloeosporioides* (94% identities) and most distant to that of *S. cerevisiae* (still 66% identities; Figure 1A). The domain prediction by the SMART website^2^ suggested that CfGcn5 contains a histone acetyltransferase acetyltransf_1 (HAT) domain and a protein binding Bromo domain (Figure 1B).

### Targeted Deletion of the *CfGCN5* Gene in *C. fructicola*

To characterize the functions of CfGcn5, the coding region of *CfGCN5* was replaced with the *HPH* gene according to the homologous recombination principle (Supplementary Figure 1A). Putative transformants were screened on hygromycin media and verified by PCR amplification. We thus acquired the *CfGCN5* gene deletion mutant Δ*Cfgcn5* (Supplementary Figure 1B). Moreover, the mutant was also complemented with the wild-type *CfGCN5* gene that restored all defects.

### CfGcn5 Is Involved in Vegetative Growth and Conidiation

**FIGURE 2:**
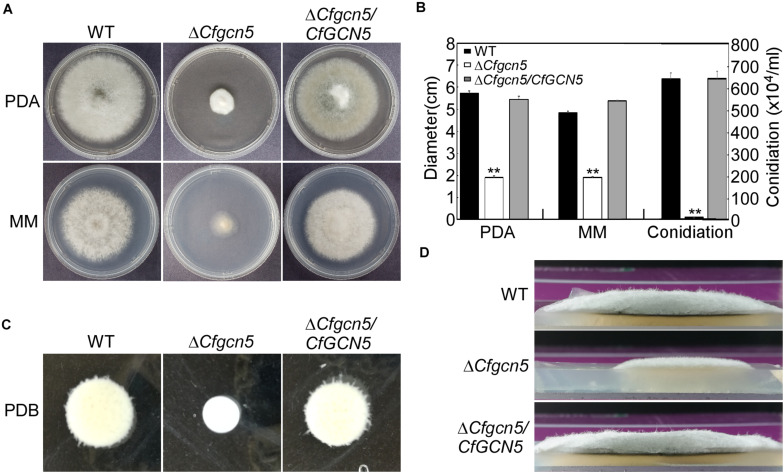
CfGcn5 is involved in vegetative growth and conidiation. **(A)** Growth of wide-type (WT), Δ*Cfgcn5* mutant, and complemented strain Δ*Cfgcn5*/*CfGCN5* on PDA and MM plates for 4 days. **(B)** Colony diameters and conidia were measured and statistically analyzed by Duncan analysis. Asterisks indicate significant differences (*p <* 0.01). **(C)** Mycelial growth following incubation in liquid PDB for 2 days. **(D)** Aerial hyphae growth is observed. Strains were cultured in PDA plates for 4 days and colony side views are displayed.

To test growth, wide-type (WT), Δ*Cfgcn5* mutant, and complemented strain Δ*Cfgcn5*/*CfGCN5* were cultured on PDA and MM plates. After 4 days, Δ*Cfgcn5* showed significant reduced growth rates compared with WT and Δ*Cfgcn5*/*CfGCN5* (Figures 2A,B). Similar results were observed following incubation in liquid PDB for 2 days (Figure 2C). Moreover, we also found that the Δ*Cfgcn5* mutant showed reduced aerial hyphal growth, which exhibited a flat colony compared with the fluffy colony of WT and Δ*Cfgcn5*/*CfGCN5* (Figure 2D). Meanwhile, we found that the Δ*Cfgcn5* mutant produced significantly fewer conidia than WT and Δ*Cfgcn5*/*CfGCN5* (Figure 2B).

### CfGcn5 Is Essential for Pathogenicity

**FIGURE 3:**
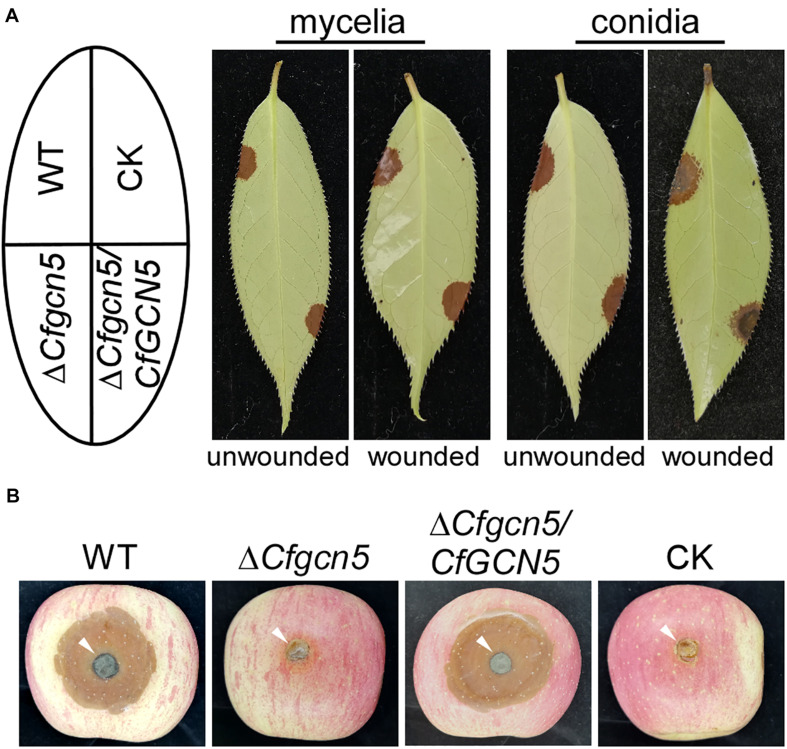
CfGcn5 is essential for pathogenicity. **(A)** Unwounded and wounded tea-oil tree leaves were inoculated with mycelial plugs or conidial suspensions of WT, Δ*Cfgcn5*, and Δ*Cfgcn5*/*CfGCN5*, respectively. Diseased symptoms were photographed at 4 days post inoculation (dpi). **(B)** Diseased symptoms of wounded apples inoculated with different mycelial plugs at 5 dpi. CK: compared control, the agar plug or ddH_2_O was inoculated onto it.

To characterize the roles of CfGcn5 in pathogenicity, mycelial plugs or conidial suspensions of WT, Δ*Cfgcn5*, and Δ*Cfgcn5*/*CfGCN5* were inoculated on unwounded tea-oil tree leaves. After 4 days, Δ*Cfgcn5* showed no lesions compared with the large and typical lesions caused by WT and Δ*Cfgcn5*/*CfGCN5* (Figure 3A). Moreover, we also conducted a pathogenicity assay on wounded tea-oil tree leaves. We found that Δ*Cfgcn5* still produced no lesions, in contrast to the typical lesions caused by WT and Δ*Cfgcn5*/*CfGCN5* (Figure 3A). Further, to test whether the non-pathogenicity of Δ*Cfgcn5* was specific to the tea-oil tree, mycelial plugs were inoculated on wounded apples, which resulted in Δ*Cfgcn5* producing no lesions and WT and Δ*Cfgcn5*/*CfGCN5* producing typical lesions (Figure 3B). The above observations indicate that CfGcn5 is essential for pathogenicity.

### CfGcn5 Is Involved in Germination and Appressoria Formation

**FIGURE 4:**
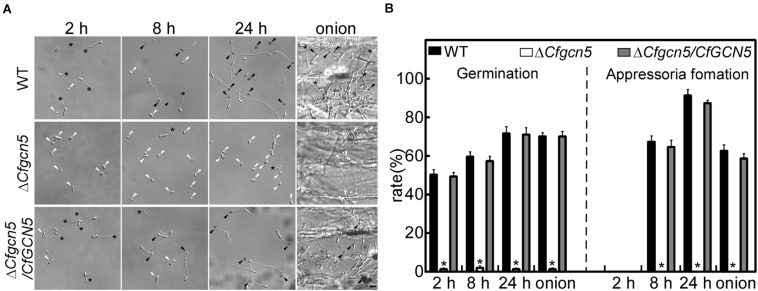
CfGcn5 is involved in germination and appressoria formation. **(A)** The conidia of WT, Δ*Cfgcn5*, and Δ*Cfgcn5*/*CfGCN5* were inoculated on artificial hydrophobic surfaces and onion epidermis, respectively. Germination and appressoria formation were observed after inoculation on hydrophobic surfaces at different time points (2, 8, and 24 h), and on onions epidermis (24 h). Asterisks indicate germ tubes, black arrows indicates appressoria, and white arrows indicates conidia. Bar = 10 μm. **(B)** Statistical analysis of germination and appressoria formation rates. Error bars represent standard deviations (SD) of three replicates and asterisks indicate significant differences (*p* < 0.01). The experiments were repeated three times with three replicates each time, and more than 100 conidia were counted per replicate.

To explore the underlying mechanism of the pathogenicity defects of the Δ*Cfgcn5* mutant, we tested conidial germination and appressoria development. After inoculation on artificial hydrophobic surfaces for 2 h, approximately 50% of conidia were germinated in WT and Δ*Cfgcn5*/*CfGCN5*, compared with 3% in the Δ*Cfgcn5* mutant (Figures 4A,B). At 8 h and 24 h, Δ*Cfgcn5* still showed less than 5% germination rates and its germ tubes did not form any appressoria, compared with more than 60% appressoria formation for WT and Δ*Cfgcn5*/*CfGCN5* (Figures 4A,B). A similar result was observed when inoculations were made on an onion epidermis, the natural appressorium-inducing hydrophobic surface. Δ*Cfgcn5* showed less than 5% germination rates and no appressoria formation at 24 h, compared with more than 60% of that for WT and Δ*Cfgcn5*/*CfGCN5* (Figures 4A,B). These results illustrate the importance of CfGcn5 in germination and appressoria formation.

### CfGcn5 Contributes to the Osmotic Stresses Tolerance

**FIGURE 5:**
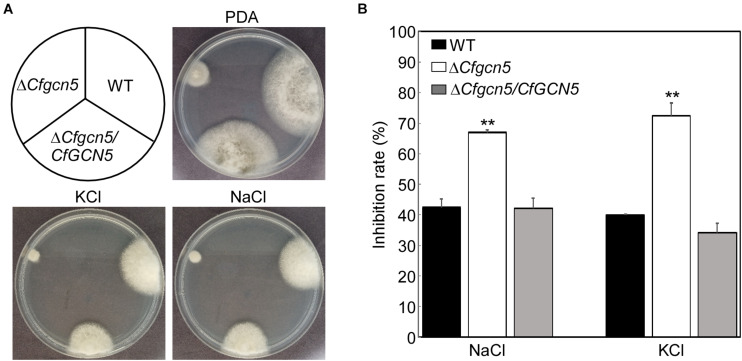
CfGcn5 contributes to the osmotic stresses tolerance. **(A)** The WT, Δ*Cfgcn5*, and Δ*Cfgcn5*/*CfGCN5* were incubated on PDA plates with various osmotic stresses of NaCl and KCl at 28°C for 4 days. **(B)** Statistical analysis of inhibition rates of the strains to osmotic stresses against untreated control. Error bars represent SD of three replicates and asterisks indicate significant differences (*p* < 0.01).

For normal growth and infection, pathogenic fungi must undergo many types of stress in nature. To address the role of CfGcn5 in environmental adaptation, WT, Δ*Cfgcn5*, and Δ*Cfgcn5*/*CfGCN5* were incubated on PDA and PDA added with osmotic stresses (1 M NaCl, and 1 M KCl) for 4 days. Δ*Cfgcn5* showed higher inhibition rates than WT and Δ*Cfgcn5*/*CfGCN5* both in NaCl and KCl (Figures 5A,B). This result indicates that CfGcn5 contributes to osmotic stresses tolerance.

### CfGcn5 Plays Roles in Cell Wall Integrity

**FIGURE 6:**
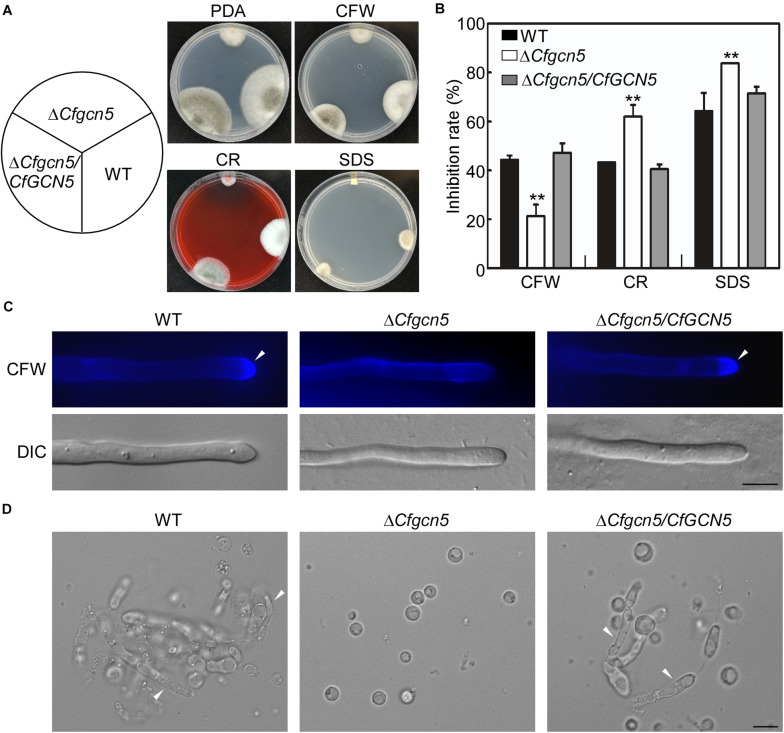
CfGcn5 plays roles in cell wall integrity. **(A)** The WT, Δ*Cfgcn5*, and Δ*Cfgcn5*/*CfGCN5* were incubated on PDA plates with various cell wall stresses of CFW, CR, and SDS at 28°C for 4 days. **(B)** Statistical analysis of inhibition rates of the strains to cell wall stresses and asterisks indicate significant differences (*p* < 0.01). **(C)** The mycelia of the strains were stained with 10 μg/mL CFW for 5 min without light before being photographed, arrows indicates the stained hyphal tips. The experiment was repeated three times with triplicates, which showed the same results. DIC, differential interference contrast image. **(D)** Light microscopic observation of protoplast release after treatment with cell wall-degrading enzymes for 2 h at 30°C, arrows indicates the non-degraded hypha. Bar = 10 μm.

We further tested the roles of CfGcn5 in the responses to cell wall stresses, namely Calcofluor white (CFW), Congo red (CR), and sodium dodecylsulfate (SDS), the results showed that Δ*Cfgcn5* exhibited lower inhibition rates in CFW and higher inhibition rates in CR and SDS than WT and Δ*Cfgcn5*/*CfGCN5* (Figures 6A,B). As CFW and CR bind to nascent chitin chains and inhibit the assembly enzymes that connect chitin to β-1,3-glucan and β-1,6-glucan (Ram and Klis, 2006), we hypothesized that CfGcn5 contributes to chitin properties. To test this, we used the CFW as a chitinous fluorochrome to stain the hyphae. In WT and Δ*Cfgcn5*/*CfGCN5*, CFW fluorescence was mostly distributed at the growing apices, where chitin, one of the main components of the fungal cell wall, was actively synthesized, while fluorescence was distributed evenly at the cell wall in Δ*Cfgcn5* (Figure 6C). We further treated the mycelia of the above strains with lytic enzymes. Mycelial fragments were still observed in WT and Δ*Cfgcn5*/*CfGCN5* after 2-h treatment, but all mycelia were degraded to protoplasts in Δ*Cfgcn5* (Figure 6D). These results revealed that CfGcn5 plays roles in maintaining cell wall integrity.

### CfGcn5 Participates in the Responses to Oxidative and ER Stresses

**FIGURE 7:**
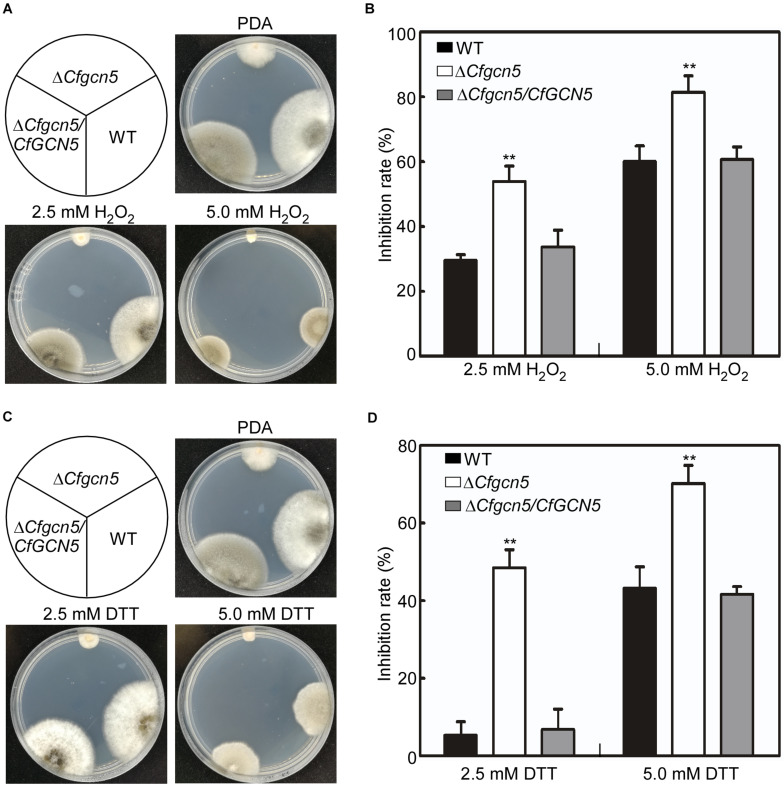
CfGcn5 participates in the responses to oxidative and ER stresses. **(A)** The WT, Δ*Cfgcn5*, and Δ*Cfgcn5*/*CfGCN5* were incubated on PDA plates with 2.5 and 5.0 mM H_2_O_2_ at 28°C for 4 days. **(B)** Statistical analysis of inhibition rates of the strains to H_2_O_2_ stress. **(C)** The WT, Δ*Cfgcn5*, and Δ*Cfgcn5*/*CfGCN5* were incubated on PDA plates with 2.5 mM DTT and 5.0 mM DTT at 28°C for 4 days. **(D)** Statistical analysis of inhibition rates of the strains to DTT stress. Error bars represent SD of three replicates and asterisks indicate significant difference (*P* < 0.01).

Apart from the environmental stresses, pathogenic fungi are also confronted with stresses from their host. The reactive oxygen species (ROS) is a common defense response for plants in pathogen–plant interactions, and pathogens must counteract host-derived ROS for successful infection (Hemetsberger et al., 2012; Liu et al., 2020). Thus, we used H_2_O_2_ to mimic host-derived ROS stress. When the strains were treated with 2.5 mM H_2_O_2_, Δ*Cfgcn5* showed an inhibition rate of 53.9%, in contrast to that of 29.5 and 33.4% in WT and Δ*Cfgcn5*/*CfGCN5*, respectively (Figures 7A,B). As the concentration of H_2_O_2_ increased, the inhibition rates of all the strains were also increased, but that of Δ*Cfgcn5* was still significantly higher than that of WT and Δ*Cfgcn5*/*CfGCN5* (Figures 7A,B). The recent studies showed that pathogens also face host-derived ER stress during infection (Tang et al., 2020; Yin et al., 2020). We thus used DTT to mimic ER stress derived from plants. When treated with 2.5 mM DTT, Δ*Cfgcn5* showed an inhibition rate of 48.5%, in contrast to that of 5.3 and 6.8% in WT and Δ*Cfgcn5*/*CfGCN5*, respectively (Figures 7C,D). A similar result was observed when the concentration of DTT was increased to 5.0 mM (Figures 7C,D). These observations revealed that CfGcn5 participates in the responses to ER stress.

### CfGcn5 Is Localized to the Nucleus

**FIGURE 8:**
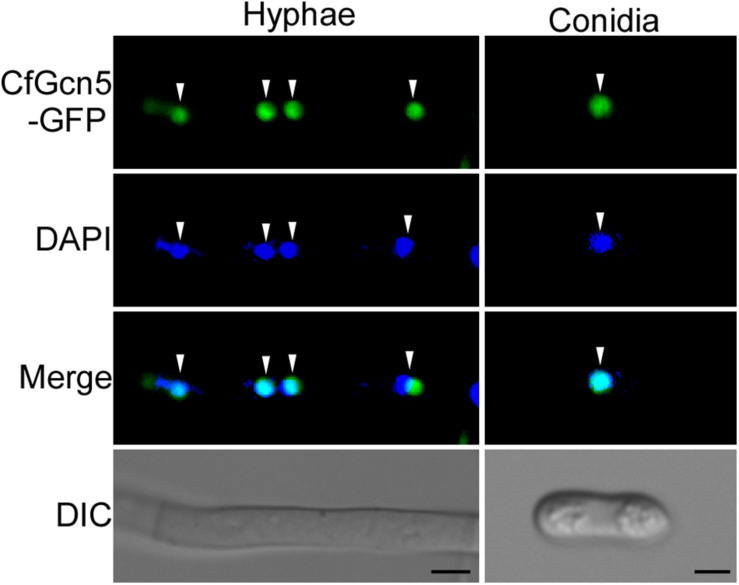
CfGcn5 is localized to the nucleus. The nuclei were stained with 4, 6-diamidino-2-phenylindole (DAPI). CfGcn5-GFP co-localized with DAPI in hyphae and conidia. Arrowheads show the representative co-localized areas. Bar = 5 μm.

To further dissect the function of CfGcn5, a green fluorescent protein (GFP) tag was fused to the C-terminus of CfGcn5 and its cellular localization was observed. Spot green fluorescent signals were observed in vegetative hyphae and conidia. Further observation by 4, 6-diamidino-2-phenylindole (DAPI, a nucleus dye) staining showed that the spot green fluorescent signals co-localized well with the blue fluorescent signals (Figure 8), indicating that CfGcn5 is localized to the nucleus.

### The NLS Contributes to the Nuclear Localization of CfGcn5

**FIGURE 9:**
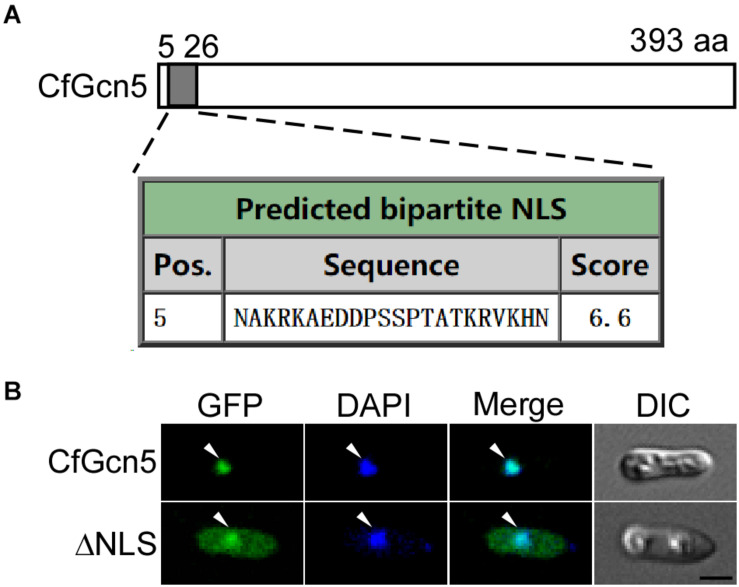
The NLS contributes to the nuclear localization of CfGcn5. **(A)** The NLS prediction of CfGcn5 with the cNLS Mapper website. **(B)** The deleted NLS region of CfGcn5 (ΔNLS) showed cytoplasmic and nuclear localization. Arrowheads show the representative co-localized areas. Bar = 5 μm.

We next predicted that CfGcn5 contains an NLS region (score > 6.0) of 5∼26 amino acids by the cNLS Mapper website (Figure 9A). We hypothesized that the localization of CfGcn5 might be mediated by the NLS region. To demonstrate this, we deleted the NLS region, and found that the truncated CfGcn5 (ΔNLS) was distributed in the cytoplasm though some still localized to the nucleus (Figure 9B). This result suggests that the NLS region mediates the nuclear localization of CfGcn5.

### The NLS Region Is Important for the Function of CfGcn5

**FIGURE 10:**
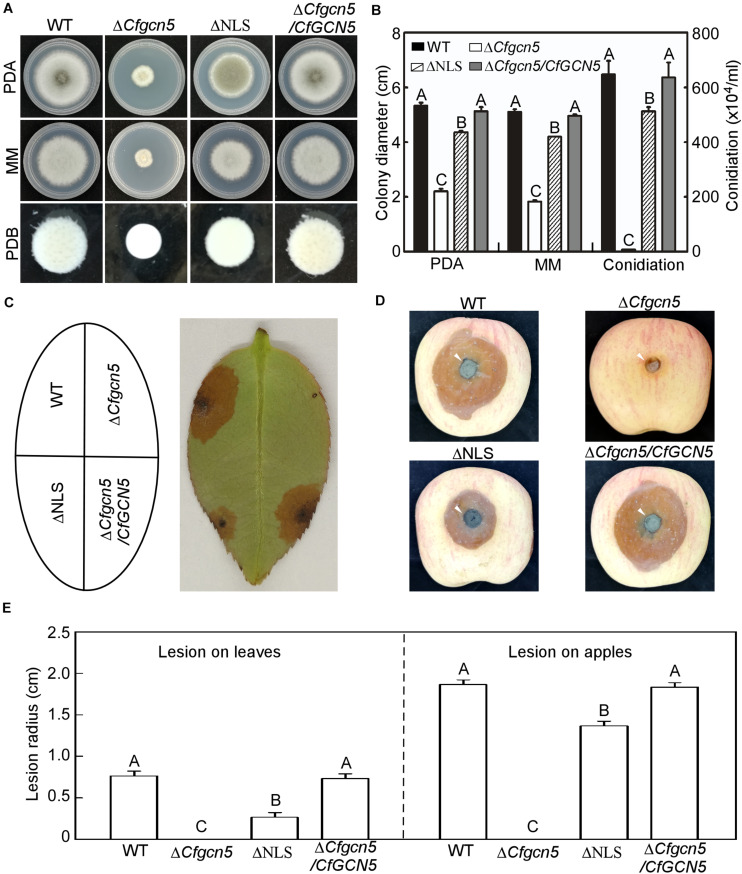
The NLS region is important for the function of CfGcn5. **(A)** Growth of WT, Δ*Cfgcn5*, ΔNLS, and Δ*Cfgcn5*/*CfGCN5* on PDA and MM plates for 4 days or in liquid PDB for 2 days. **(B)** Colony diameters and conidiation were measured and statistically analyzed by Duncan analysis. Different letters indicate significant difference (*P* < 0.01). **(C)** Diseased symptoms of wounded tea-oil tree leaves inoculated with related mycelial plugs at 4 dpi. **(D)** Diseased symptoms of wounded apples inoculated with related mycelial plugs at 5 dpi. Arrows indicate the infection area. **(E)** Statistical analysis of lesion radius on tea-oil tree leaves and apples. Error bars represent SD of three replicates and different letters indicate significant difference (*P* < 0.01).

In addition to examining the role of the NLS region in localization, we examined its contribution to function. In a growth and conidiation test, ΔNLS showed moderate growth and conidiation rates between that of Δ*Cfgcn5* and WT (Figures 10A,B). In wounded tea-oil tree leaves, ΔNLS showed some lesions but fewer than WT, while Δ*Cfgcn5* showed no lesions (Figures 10C,E). Similar results were observed in wounded apples where ΔNLS showed some lesions but still fewer than WT (Figures 10D,E). These results suggest that the NLS region is important for the function of CfGcn5.

### The HAT Domain Is Required for the Localization and Function of CfGcn5 and Bromo Domain Contributes to Its Function

**FIGURE 11:**
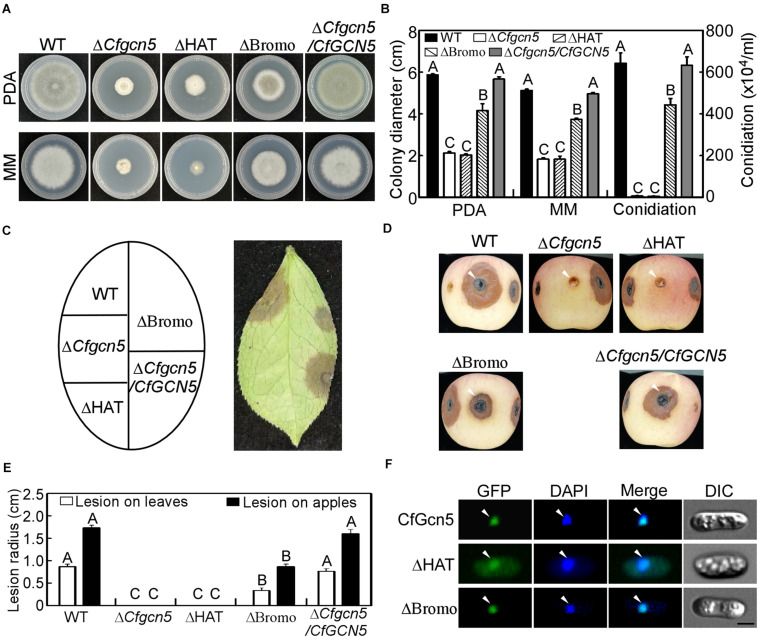
Functional and localized analysis of HAT and Bromo domains. **(A)** Growth of WT, Δ*Cfgcn5*, ΔHAT, ΔBromo, and Δ*Cfgcn5*/*CfGCN5* on PDA and MM plates for 4 days. **(B)** Colony diameters and conidiation were measured and statistically analyzed by Duncan analysis for different strains. Different letters indicate significant difference (*P* < 0.01). **(C)** Diseased symptoms of wounded tea-oil tree leaves inoculated with related mycelial plugs at 4 dpi. **(D)** Diseased symptoms of wounded apples inoculated with related mycelial plugs at 5 dpi. Arrows indicate the infection area. **(E)** Statistical analysis of lesion radius on tea-oil tree leaves and apples. Error bars represent SD of three replicates and different letters indicate significant difference (*P* < 0.01). **(F)** The localization of deleted HAT domain (ΔHAT) and Bromo domain (ΔBromo). Arrows show the representative co-localized areas. Bar = 5 μm.

Except the NLS region, CfGcn5 contains the HAT and Bromo domains. To test their contribution to function, we deleted the HAT and Bromo domains and introduced them into the Δ*Cfgcn5* mutant. We found that ΔHAT failed to restore the Δ*Cfgcn5* mutant in growth and conidiation, while ΔBromo partially restored these phenotypes (Figures 11A,B). In wounded tea-oil tree leaves, ΔHAT produced no lesions like Δ*Cfgcn5*, while ΔBromo produced some lesions but fewer than WT (Figures 11C,E). A similar pathogenic result was also observed in wounded apples (Figures 11D,E). In the localization assay, ΔHAT was distributed in the cytoplasm though some still localized to the nucleus, while ΔBromo still localized to the nucleus (Figure 10F). These results suggested that both HAT and Bromo domains contribute to the function of CfGcn5, but only the HAT domain is required for subcellular localization.

## Discussion

In the present study, we identified a yeast histone acetyltransferase ScGcn5 homolog CfGcn5 and characterized its functions. We found that the nucleus-localized CfGcn5 is important in growth, conidiation, stress responses, and pathogenicity in *C. fructicola*.

In *U. maydis* and *A. nidulans*, targeted *GCN5* gene deletion mutants showed a slight reduction in growth rate (Canovas et al., 2014; Gonzalez-Prieto et al., 2014), while that of mutants exhibited severe growth defects in *F. graminearum* and *M. oryzae* (Zhang et al., 2017; Kong et al., 2018). Similar to the growth phenotypes of these fungi, we found that the Δ*Cfgcn5* mutant showed a significantly decreased colony size and aerial hyphal growth. These observations indicate that Gcn5 proteins share conserved roles in growth, to a distinct extent.

There are some contradictions about the roles of such proteins in conidiation in other systems. In *M. oryzae*, MoGcn5 repressed conidia and the *MoGCN5* deletion mutant showed largely increased conidiation both in the light and dark conditions (Zhang et al., 2017), while such proteins were essential for conidiation in *T. reesei* and *F. graminearum*, which resulted in the mutants producing no conidia (Xin et al., 2013; Kong et al., 2018). The strongly decreased conidiation of the Δ*Cfgcn5* mutant in our study indicated that CfGcn5 is important but not essential for conidiation, which is also supported by the finding in *A. nidulans* (Reyes-Dominguez et al., 2008). These diversities in conidiation by the *GCN5* mutants reflect different mechanisms of Gcn5 proteins during asexual development in distinct fungi.

A number of external stresses that mimic the diverse micro-environments on the tea-oil tree were adapted in this study. The Δ*Cfgcn5* mutant does not only affect the resistance to osmotic stresses and cell wall integrity stresses, but is also sensitive to mimic host-derived ROS and ER stresses. The important role of CfGcn5 in the responses to environmental stress might foretell its function in virulence. Indeed, the Δ*Cfgcn5* mutant showed non-pathogenicity on healthy tea-oil leaves, which is mainly caused by the decreased conidial germination rate and no appressoria formation. Furthermore, the lack of lesions of the mutant on wounded tea-oil trees and apples indicate that there are other underlying mechanisms besides the appressorium formation, and further studies are warranted to confirm this.

Previous studies revealed that Gcn5 proteins target histones in the nucleus to epigenetically regulate global gene transcription (Chang et al., 2015; Gaupel et al., 2015; Zhang et al., 2017). We also found that CfGcn5 is localized to the nucleus both in hyphae and conidia, suggesting that nucleus localization is critical for CfGcn5 function. Both cytoplasm and nucleus localization of the deleted N-terminal NLS region indicate its contributions to localization, supported by the studies in *Candida albicans* (Chang et al., 2015). Again, the HAT domain-deleted CfGcn5 also localized in both the cytoplasm and nucleus, which shows some differences to cytoplasm but not nucleus localization of such truncated proteins in *Candida albicans*. One reason for this is that the importance of HAT in localization is distinct in different cells, and a similar result was observed in human cells when HAT-deleted Gcn5 proteins were distributed between the nucleus and cytoplasm at varying levels in different cell types (Blanco-Garcia et al., 2009). Taken together, we concluded that the HAT domain also contributes to the localization of CfGcn5, but the Bromo domain plays no role in its localization.

Combining the localization and phenotype together, we revealed that nucleus localization is essential but not sufficient for the full function of CfGcn5. The deleted NLS and HAT affected its localization, and these truncated proteins did not fully rescue the defects of the Δ*Cfgcn5* mutant when reintroduced. The deleted Bromo did not affect nucleus localization and also could not fully restore the defects of the Δ*Cfgcn5* mutant when reintroduced. These findings demonstrated that the NLS region and HAT and Bromo domains are all required for the normal function of CfGcn5. Among them, HAT may be the most important domain, as the HAT-deleted mutant showed similar defects to the Δ*Cfgcn5* mutant. This might be because HAT is essential for histone acetyltransferase activity and auto-acetylation (Candau et al., 1997; Santos-Rosa et al., 2003). It might be very interesting to investigate how acetylation occurs and how this post-translation modification regulates the pathogenicity in *C. fructicola* and other phytopathogens, such further studies are highly warranted.

## Data Availability Statement

The original contributions presented in the study are included in the article/Supplementary Material, further inquiries can be directed to the corresponding author/s. The data presented in the study are deposited in the NCBI (accession number MW701426).

## Author Contributions

HL and SZ conceived and designed the study. YG, SC, and SZ performed the experiments. SZ wrote the manuscript. All authors contributed to the article and approved the submitted version.

## Conflict of Interest

The authors declare that the research was conducted in the absence of any commercial or financial relationships that could be construed as a potential conflict of interest.

## Footnotes

1 https://www.ncbi.nlm.nih.gov/

2 http://smart.embl-heidelberg.de/

3 http://nls-mapper.iab.keio.ac.jp/cgi-bin/NLS_Mapper_form.cgi

## Supplementary Material

Supplementary Figure 1 Generation of the *CfGCN5* gene deletion mutant in *C. fructicola*. **(A)** Schematic illustration for deletion strategy of *CfGCN5* gene. **(B)** Validation of the gene deletion mutant by PCR amplified with primers 1 (NBF/NBR) and primers 2 (BWF/HPHR). M: marker; +: positive control; -: negative control; #6: mutant.

Supplementary Table 1 Primers used in this study.
