# Supplementary figures and images for: The Histone Acetyltransferase CfGcn5 Regulates Growth, Development, and Pathogenicity in the Anthracnose Fungus Colletotrichum fructicola on the Tea-Oil Tree
Source: Front Microbiol. 2021 Jun 23;12:680415. doi: 10.3389/fmicb.2021.680415 (PMC8260702; doi:10.3389/fmicb.2021.680415)

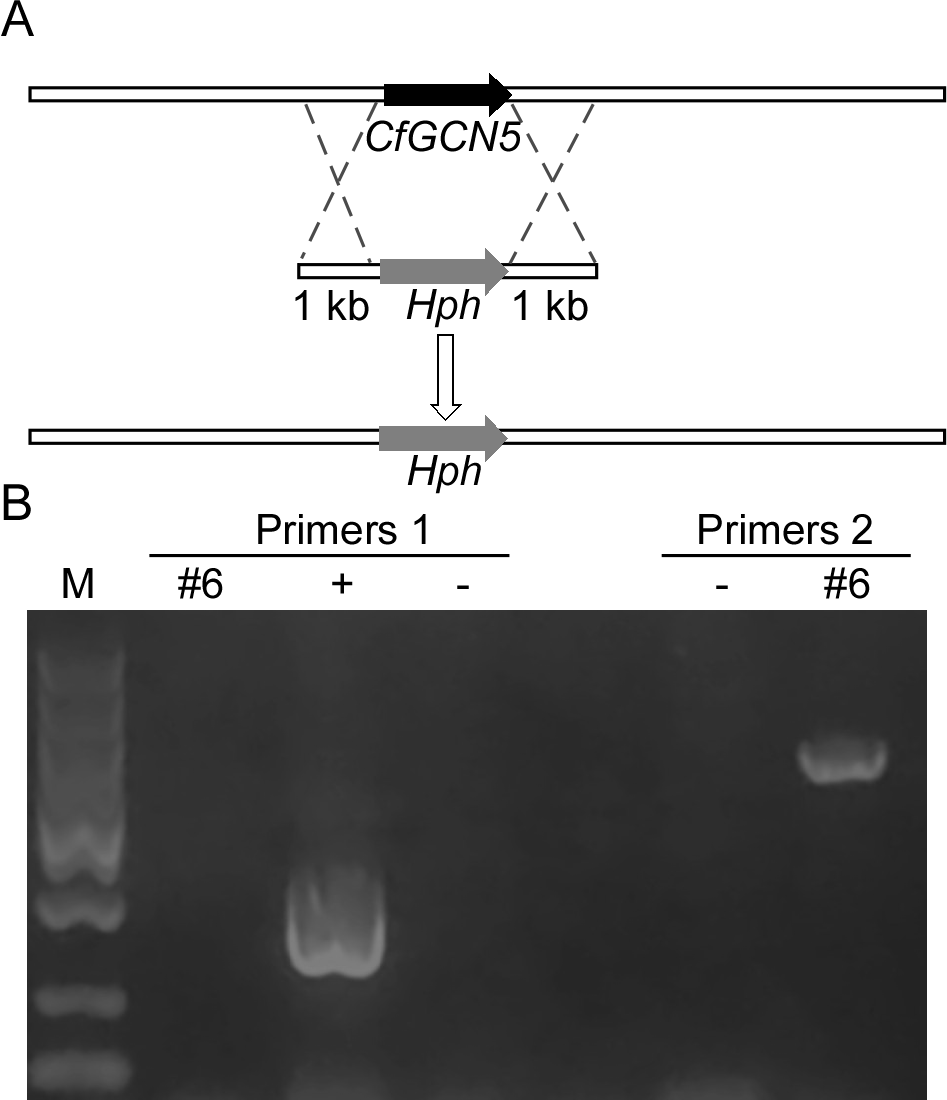

Supplement: Supplementary Figure 1 — Generation of the CfGCN5 gene deletion mutant in C. fructicola. (A) Schematic illustration for deletion strategy of CfGCN5 gene. (B) Validation of the gene deletion mutant by PCR amplified with primers 1 (NBF/NBR) and primers 2 (BWF/HPHR). M: marker; +: positive control; -: negative control; #6: mutant. [file Image_1.TIF]
