# Supplementary material for: The Histone Acetyltransferase CfGcn5 Regulates Growth, Development, and Pathogenicity in the Anthracnose Fungus Colletotrichum fructicola on the Tea-Oil Tree
Source: Front Microbiol. 2021 Jun 23;12:680415. doi: 10.3389/fmicb.2021.680415 (PMC8260702; doi:10.3389/fmicb.2021.680415)
Supplement: Supplementary Table 1 — Primers used in this study. [file Table_1.DOC]

**Table S1. Primers used in this study**

| **Primer name** | **Sequence (5’-3’)** | **Purpose** |
| --- | --- | --- |
| UF  UR  DF  DR  HYGF  HYGR  NBF  NBR  BWF  HPHR  pYF11F  pYF11R  ΔNLSR  ΔNLSF  ΔHATR  ΔHATF  ΔBromoR | TAGCTGGACATGGACTTTGTTG  TTGACCTCCACTAGCTCCAGCCAAGCCTTCGACCTAAGTAGCGAGCCTTAAC  CAAAGGAATAGAGTAGATGCCGACCGAAAGGCGGGGGAGTTCCACCGCGAA  ATGCCAGGGAGCGCAGAGGC  GGAGGTCAACACATCAATG  CTCTATTCCTTTGCCCTCG  ATGAGTCGGCAGAGCCAGAA  TGTCGGCGTATGTGAGGAAGT  GACGCCCCCTTCCTCAATC  GCTGATCTGACCAGTTGC  ACTCACTATAGGGCGAATTGGGTACTCAAATTGGTTCTGGACGGTAATTGCGGATGT  CACCACCCCGGTGAACAGCTCCTCGCCCTTGCTCACCGGTTCGAGGTGAGACCACTC  TTCTTCCTTCATTTCGACCT  GTCGAAATGAAGGAAGAAGAGTCGGCAGAGCCAGAAAAGAAGC  GAGGCCGGTGAGGATGATGA  TCATCATCCTCACCGGCCTCACTAAGGAGATCACGCTCGA  CACCACCCCGGTGAACAGCTCCTCGCCCTTGCTCACGGCGAGCTCGTCCATGTCGG | amplify *CfGCN5* 5’ flank sequence  amplify *CfGCN5* 5’ flank sequence  amplify *CfGCN5* 3’ flank sequence  amplify *CfGCN5* 3’ flank sequence  amplify *HPH* sequence  amplify *HPH* sequence  amplify *CfGCN5* gene sequence  amplify *CfGCN5* gene sequence  validation of *CfGCN5* gene deletion  validation of *CfGCN5* gene deletion  amplify complemented sequence  amplify complemented sequence  amplify ΔNLS sequence  amplify ΔNLS sequence  amplify ΔHAT sequence  amplify ΔHAT sequence  amplify ΔBromo sequence |
